# A high-throughput approach for quantifying turgor loss point in grapevine

**DOI:** 10.1186/s13007-024-01304-1

**Published:** 2024-11-24

**Authors:** Adam R. Martin, Guangrui Li, Boya Cui, Rachel O. Mariani, Kale Vicario, Kimberley A. Cathline, Allison Findlay, Gavin Robertson

**Affiliations:** 1 Department of Physical and Environmental Sciences, University of Toronto Scarborough, Toronto, Canada; 2 Horticultural & Environmental Sciences Innovation Centre, Niagara College, Welland, Canada

**Keywords:** Drought tolerance traits, Intraspecific trait variation, High throughput phenotyping, Turgor loss point, *Vitis vinifera*

## Abstract

Quantifying drought tolerance in crops is critical for agriculture management under environmental change, and drought response traits in grape vine have long been the focus of viticultural research. Turgor loss point (*π*_tlp_) is gaining attention as an indicator of drought tolerance in plants, though estimating *π*_tlp_ often requires the construction and analysis of pressure-volume (P-V) curves which are very time consuming. While P-V curves remain a valuable tool for assessing *π*_tlp_ and related traits, there is considerable interest in developing high-throughput methods for rapidly estimating *π*_tlp_, especially in the context of crop screening. We tested the ability of a dewpoint hygrometer to quantify variation in *π*_tlp_ across and within 12 clones of grape vine (*Vitis vinifera* subsp. *vinifera*) and one wild relative (*Vitis riparia*), and compared these results to those derived from P-V curves. At the leaf-level, methodology explained only 4–5% of the variation in *π*_tlp_ while clone/species identity accounted for 39% of the variation, indicating that both methods are sensitive to detecting intraspecific *π*_tlp_ variation in grape vine. Also at the leaf level, *π*_tlp_ measured using a dewpoint hygrometer approximated *π*_tlp_ values (*r*^2^ = 0.254) and conserved *π*_tlp_ rankings from P-V curves (Spearman’s *ρ* = 0.459). While the leaf-level datasets differed statistically from one another (paired *t*-test *p* = 0.01), average difference in *π*_tlp_ for a given pair of leaves was small (0.1 ± 0.2 MPa (s.d.)). At the species/clone level, estimates of *π*_tlp_ measured by the two methods were also statistically correlated (*r*^2^ = 0.304), did not deviate statistically from a 1:1 relationship, and conserved *π*_tlp_ rankings across clones (Spearman’s *ρ* = 0.692). The dewpoint hygrometer (taking ∼ 10–15 min on average per measurement) captures fine-scale intraspecific variation in *π*_tlp_, with results that approximate those from P-V curves (taking 2–3 h on average per measurement). The dewpoint hygrometer represents a viable method for rapidly estimating intraspecific variation in *π*_tlp_, and potentially greatly increasing replication when estimating this drought tolerance trait in grape vine and other crops.

## Background

Increases in the prevalence and duration of drought events due to climate change are a major concern facing agricultural production, with potentially destabilizing consequences for food security and sustainability at local through to global scales [1–3]. As a result, managing and predicting crop yield under drought is among the most pressing goals for agricultural management under environmental change, and has been for decades [4–9]. In aiming to understand the mechanistic basis of yield change under drought, one primary theme in crop science has been to better understand how plants cope with periods of water stress [10, 11].

Several reviews exist that summarize the myriad short- and long-term drought tolerance mechanisms and strategies in plants globally, which spans from short-term physiological responses such as biochemical signaling that triggers stomatal closure, through to longer-term responses including changes in leaf-, root-, or whole-plant traits [2, 3, 12–15]. Indeed, this deep literature demonstrates how and why drought tolerance represents among the most complex and multifaceted plant characteristics, and is dictated by the cumulative expression of multiple biochemical, anatomical, architectural, and morphological traits [13]. Owing to its complex nature, studies often use different approaches or metrics to quantify drought tolerance. These metrics utilize various functional traits including instantaneous water-use efficiency (measured as a ratio of photosynthetic carbon gain [*A*] to water loss through transpiration [*E*] [e.g., 16]), stomatal traits including conductance (*g*_s_) and sensitivity [e.g., 17, 18], traits associated with root phenotypes [e.g., 19], among others [e.g., 20].

One trait employed to characterize drought tolerance in plants is the turgor loss point (*π*_tlp_), which represents the leaf water potential (Ψ_leaf_) at which wilting occurs [21–23]. This trait is widely considered an indicator of drought tolerance, as plants expressing a more negative *π*_tlp_ are able to maintain leaf turgor and physiological functioning including sustained rates of *g*_s_, *A*, and *E*, across a wider range of moisture availability [23]. In the field of comparative plant and functional trait ecology, turgor loss point is considered a “higher-level” drought tolerance trait that can be used to infer leaf- and plant-level environmental tolerances [22]. As such, *π*_tlp_ and its plasticity have been employed to characterize drought tolerance across large groups of species [21, 22, 24, 25]. In turn, in studies on unmanaged ecosystems, inter- and intraspecific variation in *π*_tlp_ is used to support hypotheses surrounding the environmental determinants of plant species distributions [26, 27] and species and community-level responses to changing water availability [e.g., 18, 24, 28].

More recently, and largely as an extension of the literature on “wild” plants, *π*_tlp_ has received attention as a hypothesized index of drought tolerance in crops. For example, in detecting variation in *π*_tlp_ within and among eight cultivars of spring wheat (*Triticum aestivum* L.), Mart et al. [29] argued that *π*_tlp_ represents a trait that could be used to rapidly screen crop drought tolerance. Employing *π*_tlp_ as a crop drought tolerance trait is relatively new, since prior to this time, there was a widespread assumption that crop physiological functioning ceased below a certain soil-based permanent wilting point [as cited by 29, 30]. Furthermore, researchers have noted that relationships between *π*_tlp_ and crop growth and yield remain an unresolved area of research, leading to uncertainties as to whether or not this trait represents a viable screening tool in breeding programs that are relevant for agricultural management [31]. Nonetheless, because *π*_tlp_ is a metric that integrates multiple aspects of osmotic adjustment in plants, quantifying *π*_tlp_ within and among crop species and varieties appears an increasingly important component of the wider literature on quantifying crop drought tolerance.

**Fig. 1:**
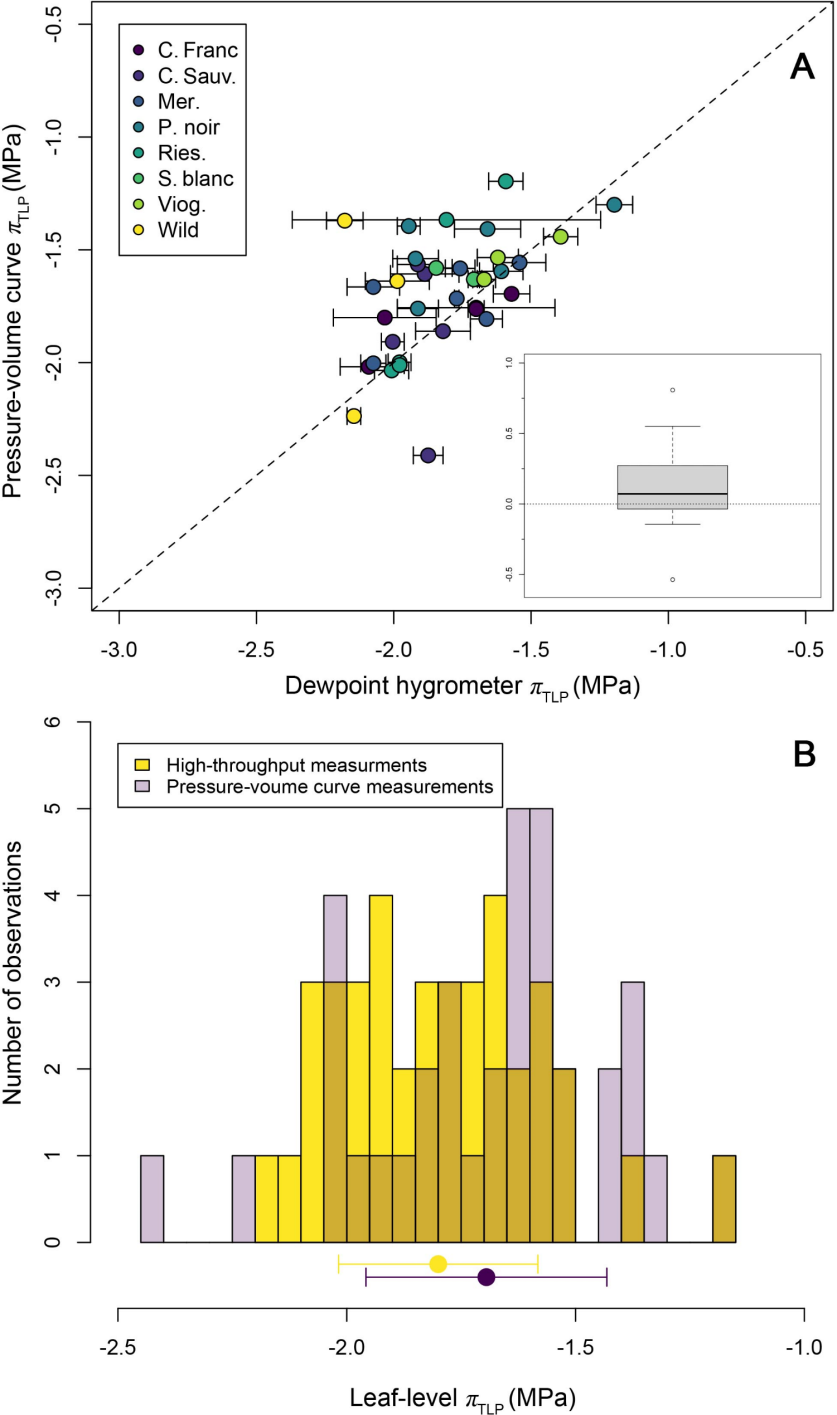
Turgor loss point (*π*_tlp_) of leaves from 12 grapevine clones and one wild relative (*Vitis riparia*) estimated through high throughput (dewpoint hygrometer) and traditional (pressure-volume curve) techniques. Panel **A** displays estimates of *π*_tlp_ derived through both methods measured on paired leaves from the same branch of individual vines. Dewpoint hygrometer-based *π*_tlp_ estimates for each individual leaf are derived as the mean of three *π*_tlp_ measurements per leaf (with error bars representing ± 1 s.e.). For clarity, points are colored according to their primary cultivar (or species in the case of the wild relative *V*. *riparia*), and the solid dashed line represents a 1:1 relationship. Inset box plot in Panel **A** presents the distribution of pairwise differences in *π*_tlp_ for all leaves in the dataset (where the average differences across *n* = 36 leaves is 0.1 MPa), such that values above 0 represent leaves where high-throughput *π*_tlp_ values are more negative than paired measurements derived through P-V curves. Panel **B** displays histograms of *π*_tlp_ values estimated with high-throughput (dewpoint hygrometer) and traditional (P-V curve) techniques. Points below the histograms correspond to the overall mean *π*_tlp_ values (± 1 s.e.) across the two methods

To date pressure-volume (P-V) curves have been the classical method for estimating *π*_tlp_ in plants, species, or genotypes [reviewed by 32], with P-V curve-derived data then supporting literature focused on plant drought tolerance and its ecological implications [33]. A single P-V curve is constructed by progressively drying a fully rehydrated leaf at set pressure or drying intervals, and then assessing the statistical relationship between Ψ_leaf_ and relative water deficit (RWD) [summarized in Fig. 1 of 22]. Data necessary for P-V curve construction and associated *π*_tlp_ estimation are generated with the use of Scholander-type pressure chambers or “pressure bombs” [34], generally following either a bench drying method where leaves are air dried on a bench top and both Ψ_leaf_ and RWD are measured at set time intervals, or a “squeeze method” where the mass of expressed sap is weighed at set pressure intervals.

Despite being widely applied in both basic and applied plant sciences for decades [34, 35], P-V curve generation is associated with major time constraints [33], often taking hours to complete for a single leaf, and certain assumptions embedded within P-V curve-based methods have motivated critical reviews on their accuracy across studies [32, 33, 36]. For example, Rodriguez-Dominguez et al. [32] elucidated how key assumptions related to sample preparation, storage, and pressurization techniques (among other factors) may lead to variability in Ψ_leaf_ measurements, and ultimately P-V curve construction from pressure chambers. At the same time, estimating *π*_tlp_ for a single leaf through a P-V curve can take hours, even when using the relatively fast squeeze method [36]. So, while P-V curves remain a critical aspect of plant and crop science, there are major limitations associated with this technique for either (1) screening drought tolerance across multiple crops and varieties, or (2) for assessing variation in drought tolerance traits at the individual plant level. The latter is especially important in the fields of crop science and agroecology, where intraspecific trait variation—i.e., variation existing below the species level—is likely a key determinant of agroecosystem processes [37].

In response to these limitations, researchers have begun developing methods for high-throughput estimation of Ψ_leaf_ and *π*_tlp_, which have gained considerable interest in studies of woody and herbaceous species in unmanaged ecosystems [33, 36, 38–40], and more recently of crops [29]. Notably, studies have employed both vapour pressure osmometers [33, 38, 39] and—the focus of our study here—dewpoint hygrometers [36, 40] to estimate *π*_tlp_ in plants by estimating this trait directly as a function of Ψ_s_ at full turgor. Specifically, a dewpoint hygrometer measures the sum of matric potential (Ψ_m_) and osmotic potential (Ψ_s_) using the chilled-mirror dewpoint technique, while the vapour-pressure osmometer measures osmolality which is then converted into Ψ_s_ following the van’t Hoff equation [33, 36]. In both these methods gravitational potential (Ψ_g_), matric potential (Ψ_m_), and pressure potential (Ψ_p_) are considered negligible when leaves are fully hydrated, and as a result Ψ_s_ in fully hydrated leaf samples can then be correlated to *π*_tlp_ [33].

The use of dewpoint hygrometers for measuring leaf bulk water relations dates back decades [41, 42] and recent studies using these techniques in high-throughput assessments of plant ecophysiology are promising. Specifically, Petruzellis et al. [40] found this method could capture interspecific variation in *π*_tlp_ across 27 Mediterranean woody species (with values ranging from ∼-4.5 to -0.5 MPa), with dewpoint hygrometer-based measurements of Ψ_s_ at full turgor linearly predicting *π*_tlp_ values derived from P-V curves (adjusted *r*^2^ = 0.46). Within species, Banks and Hirons [36] found this technique was able to quantify fine-scale differences in *π*_tlp_ that exists among five *Acer* genotypes, with mean *π*_tlp_ ranging between roughly  -1.5 to -2.0 MPa: differences that were masked when *π*_tlp_ was measured using P-V curves alone. While these studies and foundational theory [41, 42] point to the dewpoint hygrometer as a viable technique for high-throughput *π*_tlp_ estimation, no studies have yet tested if this technique is able to quantify variation in *π*_tlp_ across crop varieties.

Grapevines (*Vitis vinifera* subsp. *vinifera*) represent among the world’s most economically important crops, with the environmental conditions necessary for grapevine production varying widely across the world’s over 6,000 varieties and clones [43–46]. Many varieties or clones require a narrow range of climatic conditions for optimum plant performance, physiological functioning, fruit quality, and yield [45, 46]. Indeed, authors have argued that grapevines are among the most sensitive to climatic shifts with projections indicating that due to alterations in water availability and temperature regimes, wine production will likely shift considerably in the future [47, 48]. Grapevine drought tolerance is an integrated characteristic comprised of multiple genetic, morphological, physiological, and phenological traits [49]. Additionally, the viability of grapevine production under a shifting climate is mediated not just by drought tolerarnce, but also by other agronomic considerations such as berry size, yield, and flavor profiles. No less, high-throughput measurements of *π*_tlp_ would be valuable towards holistic prediction and modelling of climatic suitability of varieties into the future [47, 50].

In this study, we assess whether or not a high-throughput technique based on the use of a dewpoint hygrometer, is able to quantify variation in *π*_tlp_ both among and within grapevine varieties and clones. To address this, we specifically compare differences in *π*_tlp_ generated through P-V curves and a dewpoint hygrometer, at both the individual leaf- and clone-scale. While the primary focus of our study is on *π*_tlp_ variation within and among 12 widely cultivated clones of grapevines, our study also includes measurements on a wild relative of grapevines, namely *Vitis riparia*, in order to assess the wider application of this technique towards quantifying *π*_tlp_ in wild crop relatives [51]. Our study was designed to address the following research questions: (1) do grapevine varieties and clones vary significantly in their *π*_tlp_, and if so, can these differences be quantified using a high-throughput technique? Then, we asked (2) which varieties or clones are most drought-tolerant as per *π*_tlp_ values, and if inferences regarding drought tolerance rankings change depending on *π*_tlp_ estimation methods? Finally, we ask (3) do cultivated grapevines differ in their *π*_tlp_ vs. wild relatives?

## Methods

### Field site and sample collection

Grapevine plant materials for this study were collected at the Niagara College Teaching Vineyard, located in Niagara-on-the-Lake, Ontario, Canada (43.1522° N, 79.1652° W). This 16.2 ha operational vineyard established in 1996 is situated within the Niagara Peninsula Appellation in Southern Ontario, Canada. Based on downscaled climate data at a 1-km^2^ resolution [52], annual average temperatures at the vineyard are 8.6 °C, with growing season temperatures of ∼ 13.2–23.6 °C, 16.5–26.4 °C, 15.9–25.4.°C, and 11.8–21.1 °C, in June, July, August, and September, respectively. The site receives an average of 949 mm of precipitation annually, with 84, 81, 81, and 95 mm of precipitation on average falling in June through September, respectively. The vineyard is situated on silty clay with imperfect drainage, overlying clay loam till and lacustrine heavy clay with poor drainage. Tile drainage is present in alternate rows and the site is not irrigated.

At the vineyard branches of 12 cultivated *Vitis vinifera* subsp. *vinifera*. clones were sampled including Riesling (clones 23 and 171), Pinot noir (clones 89 and 828), Merlot (clones 384 and 181), Cabernet Sauvignon (clone 29 and 412), Cabernet Franc (clone 327 and 314), Viognier (clone 642), and Sauvignon blanc (clone 906). All vines are grafted to rootstock 3309, except Riesling clone 23 (rootstock SO4) and Viognier clone 642 (rootstock 101 − 14), and trained using a 2-arm flat vertical shoot position system. Our study also sampled a wild relative of cultivated grapevines *V. riparia* growing in a forest edge ecosystem situated immediately adjacent to the vineyard (i.e., within ∼ 10 m south of the vine rows at the south end of the vineyard). From each clone and wild relative species, one shoot was sampled from three different individual vines. Each shoot was ∼ 30–50 cm in length and included a minimum of three oppositely arranged leaves that were fully expanded, visually healthy, and of similar size and vigor. All leaves and shoots were collected between 9:00–10:00 over a 10-day period (July 4–14, 2023), during which there were no major precipitation events (2.3 mm of precipitation total falling on July 8 [1.1 mm], July 12 [1.6 mm], and July 13 [1.1 mm]).

Once collected, all shoots were immediately recut underwater to avoid desiccation before being transported to the lab at the University of Toronto Scarborough, Canada, which occurred within 2 h of field sample collection. In the lab, shoots were again recut under water at least 1 cm from the base and stored in the dark for 12 h to fully rehydrate. Following this step, two adjacent leaves in the same growth conditions from each branch were then used for estimating *π*_tlp_ following two different methods: (1) P-V curves executed using a SAPS II portable plant water status console (Soilmoisture Equipment Corp., CA, USA), and (2) direct measurements of Ψ_s_ at full turgor and *π*_tlp_ using a WP4C dewpoint hygrometer (METER Environmental, WA, USA).

### Estimating *π*_tlp_ using pressure-volume curves

All P-V curves were generated via the pressure chamber method following protocols described by Banks and Huron [36] and Sack et al. [53]. First, we removed one leaf from each rehydrated branch, weighed each leaf for fresh mass (FM), and recorded leaf area using an LI-3600 C leaf area meter (LICOR Bioscience, Lincoln, NE, USA). Then a cut was made at the base of the petiole using a razor blade, and the leaf was immediately weighed and sealed in the pressure chamber. The chamber was then pressurized until an initial balance pressure was reached (≥ 0.2 MPa) which was determined by the first appearance of sap expressed from the cut surface viewed under a digital microscope affixed to the SAP II console. The expressed sap was then collected using pre-weighed low-lint absorbent tissue paper inside a 1.5 ml Eppendorf tube. To prevent evaporation the opening time of the tube was minimized during sap collection. The tube and tissue paper were re-weighed after sap collection to determine the weight of water exuded. The pressure then was increased in 0.2 MPa intervals, and the sap collection procedure was repeated at least 10 times to obtain enough data points to construct a full P-V curve. In sum, this procedure took approximately 2–3 h for each individual leaf.

Based on this data, we used a series of functions in the ‘pvcurveanalysis’ R package [54] to estimate *π*_tlp_ for each leaf. First, we used the ‘FMSaturated’ function to estimate saturated fresh mass (FMs) for each leaf, which is calculated as an extrapolation of a linear regression model fit between leaf fresh mass (FM) and Ψ_leaf_ values above the estimated *π*_tlp_. Based on this FMs estimate, we used the ‘RelativeWaterDeficit” function to calculate the RWD at each pressure interval as:

1 $$RWD = 100 - 100*\left( {\left( {FM - DM} \right)\left( {FMs - DM} \right) - 1} \right)$$

where DM is dry mass measured at the end of each P-V curve by drying each leaf at 65 °C to constant mass, and FMs is FM at water saturation. Then, *π*_tlp_ was estimated for each leaf using the ‘OsmoticPot’ function in the ‘pvcurveanalysis’ R package [54].

### Estimating *π*_tlp_ using a dewpoint hygrometer

For high throughput estimates of *π*_tlp_ we used the WP4C dewpoint hygrometer to measure Ψ_s_ at full leaf turgor (with plants being rehydrated as described above), and subsequently convert this value to *π*_tlp_ estimates. Our methods followed those described by previous studies using dewpoint hygrometers in estimating *π*_tlp_ from Ψ_s_ at full leaf turgor values, based on rehydrated shoots/ leaves [36, 40]. In our analyses, leaves immediately adjacent to those used in P-V curve analyses were selected, and we collected three leaf discs per leaf (or pseudo-replicates) that were 35 mm in diameter from the base of each lobe using a circle cutting blade. Leaf discs were immediately wrapped in tinfoil to avoid water loss, flash frozen in liquid N_2_ for five minutes, and then gently abraded using 600 grit sandpaper to remove the cuticle before being placed in the dewpoint hygrometer chamber. Measurements of Ψ_s_ at full turgor were collected using the WP4C continuous reading mode and recorded after 10–15 min when the measuring chamber reached vapor equilibrium. The dewpoint hygrometer was calibrated prior to every 10 measurements using 0.5 mol KCl solution. Based on values of Ψ_s_ at full turgor, we then estimated *π*_tlp_ following the model described by Bartlett et al. [33] as:

2 $${\pi _{tlp}} = \left( {{\Psi _s}*0.832} \right) - 0.631$$

### Statistical analysis

Statistical analysis and data visualization were performed using R v. 4.2.2 statistical software (R Foundation for Statistical Computing, Vienna, Austria). Our first analysis made use of our dataset that included a total of *n* = 39 measurements of *π*_tlp_ estimated using both P-V curves and the dewpoint hygrometer, such that all leaf-level dewpoint hygrometer *π*_tlp_ were calculated as the mean of three *π*_tlp_ pseudo-replicates per leaf. Using this data, we first performed a *t*-test to evaluate differences in leaf-level *π*_tlp_ across the two datasets, and followed with a linear regression model (where *n* = 39 leaves) to test whether *π*_tlp_ derived from the high-throughput method predicts *π*_tlp_ derived from P-V curves. Additionally, we also used a linear hypothesis test implemented in the ‘car’ R package [55] to compare this linear regression model with a 1:1 relationship where *π*_tlp_ from the high-throughput method perfectly corresponds to *π*_tlp_ from P-V curves. This linear regression and hypothesis test analysis was supplemeted with a Spearman rank correlation test, to evaluate the degree to which rankings of *π*_tlp_ change as a function of methodology.

Using this same leaf-level dataset, we fitted mixed effects models coupled with variance partitioning techniques to quantify the proportion of variation in *π*_tlp_ values that were attributable to variety identify (e.g., Riesling vs. Cabernet Franc), clone identity (e.g., Riesling clones 23 vs. 171), and methodology (i.e., dewpoint hygrometer vs. P-V curves). Due to sample size limitations, we performed this analysis by fitting a series of mixed models vs. using a single model. Specifically, we used the ‘lme’ function in the ‘nlme’ R package [56] to fit mixed effects models that predicted variation in *π*_tlp_ as a function of: (1) clone identity as a fixed effect and methodology as a random effect; (2) methodology as a fixed effect and clone identity as a random effect; and (3) variety identity as a fixed effect and methodology as a random effect. For each of these models we applied the ‘r.squaredGLMM’ function in the ‘MuMIn’ R package [57] to estimate the proportion of variation in *π*_tlp_ associated with both fixed effects (or the marginal *r*^2^ values) and fixed and random effects combined (or the conditional *r*^2^ values) [58].

For additional analysis, we replicated our leaf-level analysis at the clone/species level. To do so, we calculated mean *π*_tlp_ values for each clone/species-by-method combination thereby generating a clone/species-level dataset based on *n* = 3 observations of *π*_tlp_ in total for each method (except in the case of Cab. Franc 314, Cab. Sauv. 412, and Riesling 23, where one P-V curve failed). Then, the same *t*-test, linear regression, linear hypothesis test, and Spearman rank correlation procedures described above were performed on this species/clone-level dataset (where *n* = 13 for each test).

## Results

Across the leaf-level dataset mean *π*_tlp_ values were-1.7 ± 0.3 (s.d.) MPa (interquartile range = 0.3 MPa) when estimated using the P-V curve method, compared to a mean *π*_tlp_ of -1.8 ± 0.2 MPa (interquartile range = 0.3 MPa) using the high throughput method (Fig. 1). Observed *π*_tlp_ values derived from P-V curves ranged from − 2.4 to -1.2 MPa, and from  -2.2 to -1.2 MPa using the dewpoint hygrometer (Fig. 1B). Differences in *π*_tlp_ for individual vines calculated as P-V curve *π*_tlp_ minus dewpoint hygrometer *π*_tlp_ values, ranged from  -0.5 MPa to 0.8 MPa and averaged 0.1 ± 0.2 (s.d.) MPa (Fig. 1). A paired *t*-test indicated that these two datasets differed statistically from one another (*t* = 2.96, d.f.=35, *p* = 0.006), though these differences owe largely to a *π*_tlp_ value from P-V curves from one *V*. *riparia* leaf that was the most negative *π*_tlp_ value in our dataset (Fig. 1A).

Spearman rank correlation analysis indicated that *π*_tlp_ rankings among individual vines was significantly correlated across methodologies (Spearman’s *ρ* = 0.5, *p* = 0.002). Similarly, a linear regression analysis found that *π*_tlp_ estimates generated by the dewpoint hygrometer explained 25.4% of the variation in *π*_tlp_ generated by P-V curves (regression model *p* = 0.002, intercept=-0.59 ± 0.32 (s.e.), slope = 0.61 ± 0.18; Fig. 1A). However, the relationship between paired *π*_tlp_ measurements did diverge significantly from a 1:1 relationship (linear hypothesis test *F* = 7.3, *p* = 0.002; Fig. 1).

**Fig. 2:**
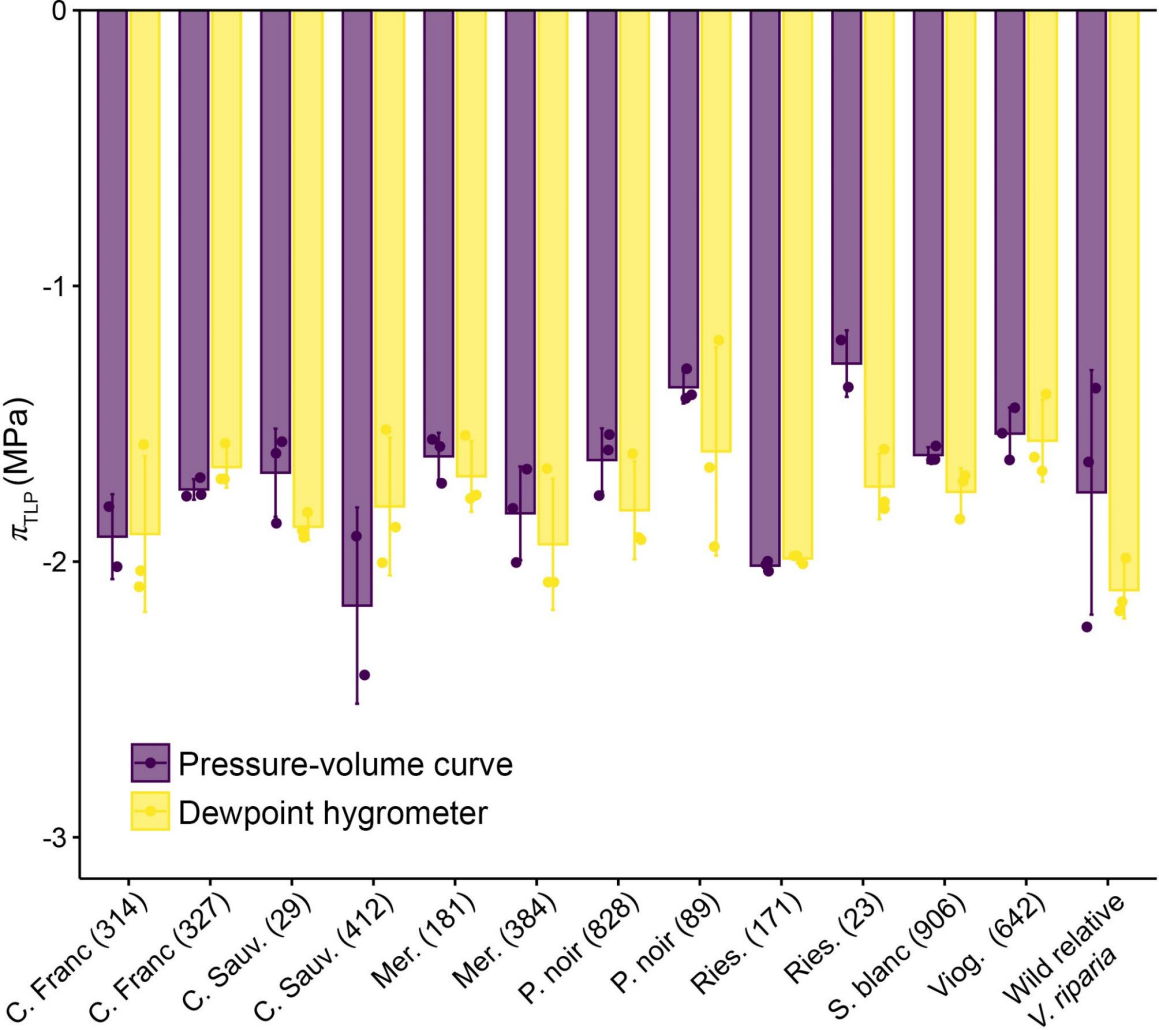
Leaf- and clone-/species-level turgor loss point (*π*_tlp_) estimates for 12 grapevine varieties and one wild relative derived through high throughput and pressure volume curves methodology. Individual points correspond to individual leaf-level observations of *π*_tlp_. Leaf-level *π*_tlp_ values associated with the dewpoint hygrometer method correspond to the mean of *n* = 3 pseudo-replicates per individual leaf, while leaf-level *π*_tlp_ values associated with pressure-volume curves are derived from one curve per leaf. Bars correspond to species/clone-level average *π*_tlp_ values (± 1 s.e.) associated with *n* = 3 measurements for each species/clone-by-method combination

Our mixed effects model analyses found that across the leaf-level dataset, clone/species identity accounted for the largest proportion of variation in *π*_tlp_ observations. Specifically, a mixed effects model that included clone/species identity as a fixed effect and method as a random effect detected statistically significant differences in *π*_tlp_ across varieties/species (*F*_12, 61_=4.32, *p* < 0.001; Fig. 2). In this model, clone/species identity explained 38.6% of the variation in *π*_tlp_ across all observations (i.e., based on the marginal *r*^2^ value), while the method (incorporated as a random effect) explained only an additional 6.0% of the variation in *π*_tlp_ (i.e., based on the conditional *r*^2^ value). These results were largely robust when leaf-level data was analyzed with method as a fixed effect (marginal *r*^2^ = 0.043) and clone as a random effect (conditional *r*^2^ = 0.394), though here method was a statistically significant predictor of *π*_tlp_ values (*F*_1, 61_=5.25, *p* = 0.025).

**Fig. 3:**
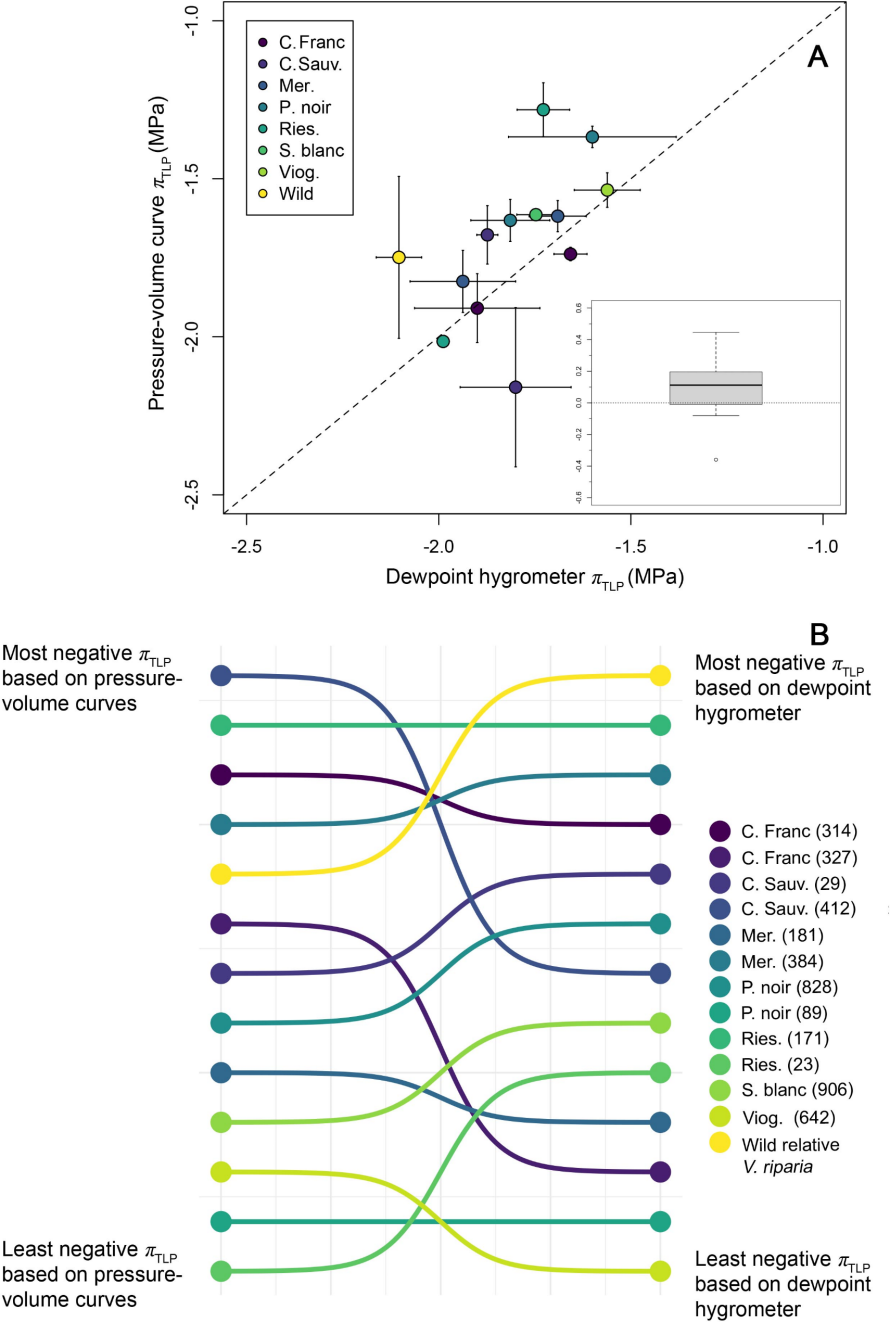
Average turgor loss point (*π*_tlp_) of 12 grapevine clones and one wild relative (*Vitis riparia*) estimated through high throughput (dewpoint hygrometer) and traditional (pressure-volume curve) techniques. Panel **A** displays estimates of species/clone-level mean *π*_tlp_ derived through both methods (with error bars representing ± 1 s.e.). The solid dashed line represents a 1:1 relationship. The inset box plot presents the distribution of pairwise differences in *π*_tlp_ for all species/clones in the dataset, such that values above 0 represent leaves where high throughput *π*_tlp_ values are more negative than paired measurements derived through pressure-volume curves. Panel **B** represents rank shifts in estimated *π*_tlp_ for species/clones when estimated using different techniques. Ordering of the left-hand side points denotes *π*_tlp_ rankings based on pressure-volume curves, while ordering of the right-hand side points denotes *π*_tlp_ rankings based on high throughput methods

When data was analyzed at the species/clone level (*n* = 13) we did not detect significant differences in mean *π*_tlp_ across methods (*t* = 1.74, d.f.=13, *p* = 0.107; Fig. 3A). Specifically, across species/clones, mean *π*_tlp_ values measured using P-V curves were − 1.7 ± 0.2 (s.d.) MPa and ranged from − 2.2 ± 0.3 (s.e.) in Cabernet Sauvignon clone 412, to -1.2 ± 0.1 (s.e.) MPa in Riesling clone 23 (Fig. 3A). When *π*_tlp_ was based on dewpoint hygrometer measurements, mean *π*_tlp_ across all varieties averaged − 1.8 ± 0.2 (s.d.) MPa, ranging from − 2.1 ± 0.1 (s.e.) in *V*. *riparia*, to -1.6 ± 0.1 (s.e.) MPa in Viognier clone 642 (Fig. 3).

The ranking of species/clones based on their mean *π*_tlp_ values did not differ statistically based on methodology (Spearman’s *ρ* = 0.69, *p* = 0.011), with the rank of a number of varieties at both the most (e.g., Riesling 171) and least negative *π*_tlp_ values (i.e., Pinot noir clone 89) being robust towards methodology (Fig. 3B). And while the ranking of certain varieties did shift across methods (e.g. Cabernet Franc clone 372), most rank changes were limited to two to three positions on a *π*_tlp_ ranking scheme (Fig. 3B). Finally, a linear regression model found that clone-level mean *π*_tlp_ values based on dewpoint hygrometer measurements explained 30.4% of the variation in *π*_tlp_ values from P-V curves (regression model *p* = 0.05, intercept=-0.18 ± 0.7 (s.e.), slope = 0.84 ± 0.39), and at the species/clone level this relationship did not differ statistically from a 1:1 relationship (linear hypothesis test *F* = 1.49, *p* = 0.267; Fig. 3A).

## Discussion

Estimating *π*_tlp_ using a dewpoint hygrometer presents a promising avenue for high-throughput assessments of grapevine drought tolerance. This technique closely approximates values of the same trait derived from traditional P-V curve techniques (Figs. 1, 2 and 3), being able to quantify relatively fine-scale variation that exists among closely related varieties and species. Our results contribute to the literature on high-throughput *π*_tlp_ estimation, which gained considerable popularity with the development of osmometry-based methods [25, 33], extending to recent analyses employing dewpoint hygrometers [36, 40, 59]. Our results align with this previous work indicating that a dewpoint hygrometer, alongside established models correlating *π*_tlp_ and Ψ_s_ at full turgor [33], support the generation and analysis of an important drought tolerance trait in plants. However, our work extends this literature to suggest high-throughput methods are equipped to capture the fine-scale variation in *π*_tlp_ that exists among individual plants or varieties of the same species: a key consideration for studies on crop functional trait variation [37].

One consistent finding in our results is that at either the leaf- or clone/species level, *π*_tlp_ values derived from the dewpoint hygrometer were on average 0.11 or 0.1 MPa lower (more negative) than paired observations from a P-V curve: values corresponding to average declines of *π*_tlp_ by 5–6% as one moves from traditional to high-throughput methods (Figs. 1 and 3). This trend is similar to results obtained by Banks and Hirons [36] in their efforts to quantify intraspecific variation in *π*_tlp_ across five maple (*Acer spp.*) genotypes, where the same hygrometer model generated more negative *π*_tlp_ values in comparison with those derived from P-V curves. Similarly, one study on a single grapevine variety also found *π*_tlp_ from a dewpoint hygrometer strongly correlated with values from P-V curves, with more negative values derived from the high-throughput technique [59].

A proposed explanation for this trend is related to methods associated with P-V curve generation. Specifically, potential water loss during petiole excision, solute accumulation in undamaged tissue, and the possibility that Ψ_leaf_ is lower than water potential in the air (i.e., conditions of high ambient humidity), could all lead to higher *π*_tlp_ estimates derived from P-V vs. dewpoint hygrometer measurements [36, 59, 60]. In our study though, absolute and relative differences in *π*_tlp_ across methods were smaller (i.e., 5–6%) compared to observations in other studies (e.g., ∼ 26.5% on average [36, 59]). No less, the growing literature on high-throughput approaches to *π*_tlp_ estimation including our own results, indicates these methods provide a viable alternative to P-V curves especially in situations where large sample sizes are an important consideration.

In relation to issues of sample size, at the leaf-level our high-throughput approach resulted in a slightly narrower range of *π*_tlp_ values (-1.2 to -2.18 MPa) compared to those generated by P-V curves (-1.2 to -2.41 MPa). Here, the larger range of *π*_tlp_ values derived from P-V curves at both the leaf- and species/clone, was in part associated with a large negative *π*_tlp_ estimated for one Cab. Sauv. clone 412 leaf (where *π*_tlp_ = -2.41 MPa). This trend then scaled-up to support patterns at the species/clone-level, where the dewpoint hygrometer method supported a more restricted range of mean *π*_tlp_ from values (-1.56 to -2.1 MPa) vs. P-V curve-based data (-1.28 to -2.16 MPa) (Fig. 3). While these differences in data ranges are relatively small, they may be associated with issues associated with P-V curve generation (noted above). Therefore, the (pseudo-)replication afforded by the high-throughput method may provide more robust *π*_tlp_ values for individual clones.

Related, the most notable is the difference in time required to generate data using these different methods. Each of the 39 P-V curves executed in our study required 2–3 h of processing time, with three of them failing; analytical steps including visual inspection of all P-V curves prior to final curve fitting further added to this time requirement. Comparatively, each of the dewpoint hygrometer values took ∼ 12–15 min to generate a single *π*_tlp_ data point. This time consideration is clearly of interest for plant and crop scientists, and indeed is the foundation of multiple high-throughput approaches to screening crop ecophysiological responses to environmental conditions [e.g., 61]. But despite the inherit value of high-throughput screening in terms of time and potential for greater (pseudo-)replication, there remain limitations. Since the WP4C model requires a leaf disc to be 35 mm, plants and crops with leaves smaller than 35 mm diameter cannot be analyzed by this instrument, and species with thicker cuticles or succulents are likely to require specialized preparation methods before measurement. Lastly, P-V curves also return highly informative metrics associated with leaf water relations, including for example estimates of the bulk elastic modulus or the apoplastic fraction: key traits contributing to plant and crop drought tolerarnce and stress responses [21, 62, 63].

## Conclusions

Crop responses to drought conditions have long been the focus of applied agricultural research, with crops showing complex responses to reduced water availability [2, 3]. These responses span above- and belowground biophysical processes, and synthetically incorporate phenology, multiple functional traits, biochemical signaling pathways, and genes across leaves, roots, stems, and reproductive structures [49, 64]. While drought represents only one part of the wider science surrounding threats to food security [65], identifying drought tolerant crops and genotypes is clearly of importance for maintaining yields under a shifting climate [66]. High-throughput estimation of functional traits associated with drought tolerarnce, both within and among crop species, varieties, and genotypes, service this goal in part. Traditional techniques associated with quantifying plant-water relations remain invaluable in estimating certain traits. Though high-throughput techniques such as those evaluated here, especially when coupled with other techniques, appear well suited and able to rapidly quantify components of drought responses in crops, at data acquisition rates that match the urgency of global change science.

## Data Availability

Upon publication, the dataset supporting the conclusions of this article will be made available upon request to the corresponding author, and in the TRY Functional Trait Database.
